# Efficacy and safety of adjuvant chemotherapy in T1N0M0 intrahepatic cholangiocarcinoma after radical resection

**DOI:** 10.1186/s12885-022-10269-0

**Published:** 2022-11-10

**Authors:** Xiao-hui Li, Chong-yu Zhao, En-liang Zhou, Xiao-jun Lin

**Affiliations:** Department of Pancreatobiliary Surgery, State Key Laboratory of Oncology in South China, Collaborative Innovation Center for Cancer Medicine, Sun Yat-sen University Cancer Center, 651 Dongfengdong Road, Guangzhou, 510060 China

**Keywords:** Adjuvant chemotherapy, Adverse reactions, Efficacy, Intrahepatic cholangiocarcinoma, Survival analysis

## Abstract

**Objective:**

Adjuvant chemotherapy is necessary for radical resection of intrahepatic cholangiocarcinoma (ICC) with a high risk of recurrence (T2–4, N1). However, its use in the treatment of early-stage ICC remains controversial. This study aimed to investigate the role of adjuvant chemotherapy after radical resection in patients with early-stage ICC (T1N0M0).

**Data and methods:**

The data of 148 patients with pathologically diagnosed ICC (T1N0M0) who underwent radical resection from January 2012 to January 2018 at the Sun Yat-sen University Cancer Center were retrospectively analyzed. Using consistent baseline data, Kaplan–Meier survival curves were constructed to compare relapse-free survival (RFS) and overall survival (OS) between patients who received postoperative adjuvant chemotherapy (AC group) and those who received only surgical treatment (non-AC group). Univariate and multivariate Cox regression analyses were used to screen for independent prognostic factors affecting survival. The RFS and OS of patients were analyzed after the administration of three adjuvant chemotherapy regimens (gemcitabine + capecitabine [GX], gemcitabine + cisplatin [GP], and capecitabine monotherapy [X]). Finally, the safety of adjuvant chemotherapy was evaluated based on the incidence of grade 1–4 adverse events.

**Results:**

The median RFS was 18 months in the non-AC group and 25 months in the AC group. The median OS was 34 months in the non-AC group; however, it was not reached in the AC group. The OS of the AC group was significantly higher than that of the non-AC group (*P* = 0.005). Multivariate Cox analysis demonstrated that nerve invasion (*P* = 0.001), preoperative elevation of cancer antigen 19–9 (CA 19–9) levels (*P* = 0.009), and postoperative adjuvant chemotherapy (*P* = 0.009) were independent prognostic factors for early-stage ICC after radical resection. The OS rates of the GX, GP, X, and non-AC groups were significantly different (*P* = 0.023) and were higher in the GX group than in the non-AC group (*P* = 0.0052). Among patients with elevated preoperative CA 19–9 levels, the OS rate was higher in the AC group than in the non-AC group (*P* = 0.022). In terms of safety, the incidence of grade 3 or 4 adverse reactions was < 18.2% in the GX, GP, and X groups, without the occurrence of death owing to such reactions.

**Conclusion:**

Adjuvant chemotherapy can prolong OS among patients with early-stage ICC who have undergone radical resection. Preoperative elevation of CA 19–9 levels and nerve invasion are independent prognostic factors for poor survival outcomes for early-stage ICC after radical resection. All chemotherapy regimens used in the study are safe.

## Introduction

Intrahepatic cholangiocarcinoma (ICC) is an adenocarcinoma originating from intrahepatic secondary bile ducts and their branched epithelial cells. According to the epidemiological data reported by the National Cancer Center in 2015, the incidence of liver cancer was 26.92 per 100,000 population in China [1]. Given that ICC accounts for 10–15% of all liver cancer cases [2], the incidence of ICC in China is approximately 2.69 per 100,000 population. Additionally, the incidence of ICC has increased worldwide in recent years [3]. ICC has a very poor prognosis, with the postoperative 5-year overall survival (OS) rate of 25–40% [4]. Adjuvant chemotherapy can increase the survival of some patients with ICC postoperatively. According to the American Society of Clinical Oncology (ASCO) [5] guidelines, patients with resected biliary tract cancer should be recommended adjuvant capecitabine for 6 months. The National Comprehensive Cancer Network (NCCN) guidelines also support the use of adjuvant chemotherapy for biliary tract cancer [6]. Schweitzer et al. [7] showed that the OS of patients who received adjuvant chemotherapy (33.5 months) was significantly better than that of patients who underwent only surgical resection (18.0 months). However, the selection of patients with ICC who can benefit from adjuvant chemotherapy remains controversial. Reames et al. [8] showed that postoperative gemcitabine chemotherapy prolonged the survival of patients with a high risk of recurrence and metastasis (patients with T2, T3, T4, and N1 disease). To date, most researchers have proposed that patients with prognostic risk factors should be selected for adjuvant chemotherapy, such as those with stage T2–4 disease, lymph node metastasis, vascular invasion, positive margin, and nerve infiltration [9–11]. However, no consensus has been reached on whether adjuvant chemotherapy is required for patients with early-stage ICC (T1N0M0). This study aimed to investigate the necessity and safety of postoperative adjuvant chemotherapy among patients with early-stage ICC.

## Data and methods

### Data sources

**Fig. 1:**
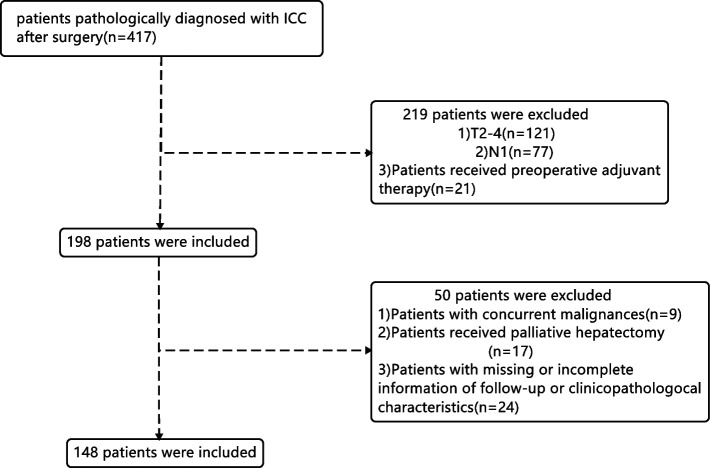
Flow chart of the patient enrolling process

Patients who were pathologically diagnosed with ICC between January 2012 and January 2018 at the Sun Yat-sen University Cancer Center were selected. The inclusion criteria were as follows: (1) pathological confirmation of ICC; (2) the presence of T1N0M0-stage disease; (3) no use of neoadjuvant radiotherapy or chemotherapy preoperatively. The exclusion criteria were as follows: (1) nonradical resection (pathologically confirmed positive resection margin); (2) perioperative death; (3) concurrent malignancies; (4) missing or incomplete information. A flow diagram for data selection is shown in Fig. 1. All procedures involving human participants were in accordance with the ethical standards of the 1964 Declaration of Helsinki and its later amendments.

### Surgical treatment and adjuvant chemotherapy

The surgical method of hepatectomy was selected according to the tumor site, relationship of the tumor with the liver and surrounding important blood vessels, liver cirrhosis, and residual liver volume. Patients included in this study received the following three postoperative adjuvant chemotherapy regimens: gemcitabine + capecitabine (GX), gemcitabine + cisplatin (GP), and capecitabine monotherapy (X). The specific dose of chemotherapeutic drugs was individually determined by a clinical oncologist and was adjusted according to the situation of the patients.

### Follow-up

Outpatient follow-up was performed 1 month postoperatively and every 3 months thereafter if no recurrence was observed. The follow-up evaluation included blood routine test, liver function test, liver tumor markers and upper abdominal computed tomography (CT). OS and relapse-free survival (RFS) were the primary endpoints.

### Statistical analysis

The SPSS Statistics and R (version 4.1.2) software were used for analysis. The Kaplan–Meier method was used to draw survival curves, and a Cox proportional hazards regression model was used for univariate and multivariate analyses. A *P*-value of < 0.05 was considered statistically significant.

## Results

### Baseline data

The demographic and clinical characteristics of patients are shown in Table 1. A total of 84 patients who received only surgical treatment were included in the non-AC group; of which, 43 were men and 41 were women, with a median age of 59 years. A total of 64 patients who received postoperative adjuvant chemotherapy were included in the AC group; of which, 40 were men and 24 were women, with a median age of 58 years. No significant difference was observed in baseline data between the two groups (all *P* > 0.05).

**Table 1:** Baseline characteristics of two groups

Variables	Postoperative adjuvant chemotherapy		X2	***P***
	Absence (***n*** = 84)	Presence(***n*** = 64)		
Gender				
male	43	40		
female	41	24	1.886	0.170
Age (years)				
< =55	19	22		
> 55	65	42	2.507	0.113
Tumor differentiation				
low	18	5		
moderate	61	56		
high	5	3	5.458	0.065
Tumor size (cm)				
> 5 cm	37	24		
< =5 cm	47	40	0.643	0.423
Microvascular.invasion				
absence	60	43		
presence	24	21	0.309	0.578
Nerve invasion				
absence	68	56		
presence	16	8	1.146	0.284
Preoperative Ca19–9(u/ml)				
≤ 37.00u/ml	37	27		
> 37.00u/ml	47	37	0.051	0.821
Preoperative TBIL (umol/L)				
≤ 17.1 umol/L	71	59		
> 17.1 umol/L	13	5	1.997	0.158
HbsAg				
positive	48	33		
negative	36	31	0.457	0.499
Surgical type				
Open surgery	61	44		
laparoscopic surgery	23	20	0.264	0.608
Type of liver resection				
local liver resection	25	26		
left lateral lobe resection	17	14		
left hemihepatectomy	16	9		
right hemihepatectomy	11	9		
Other types of liver resection	15	6	3.692	.449
Postoperative molecular targeted drug				
absence	67	49		
presence	17	15	0.219	0.639

### Survival analysis

**Fig. 2:**
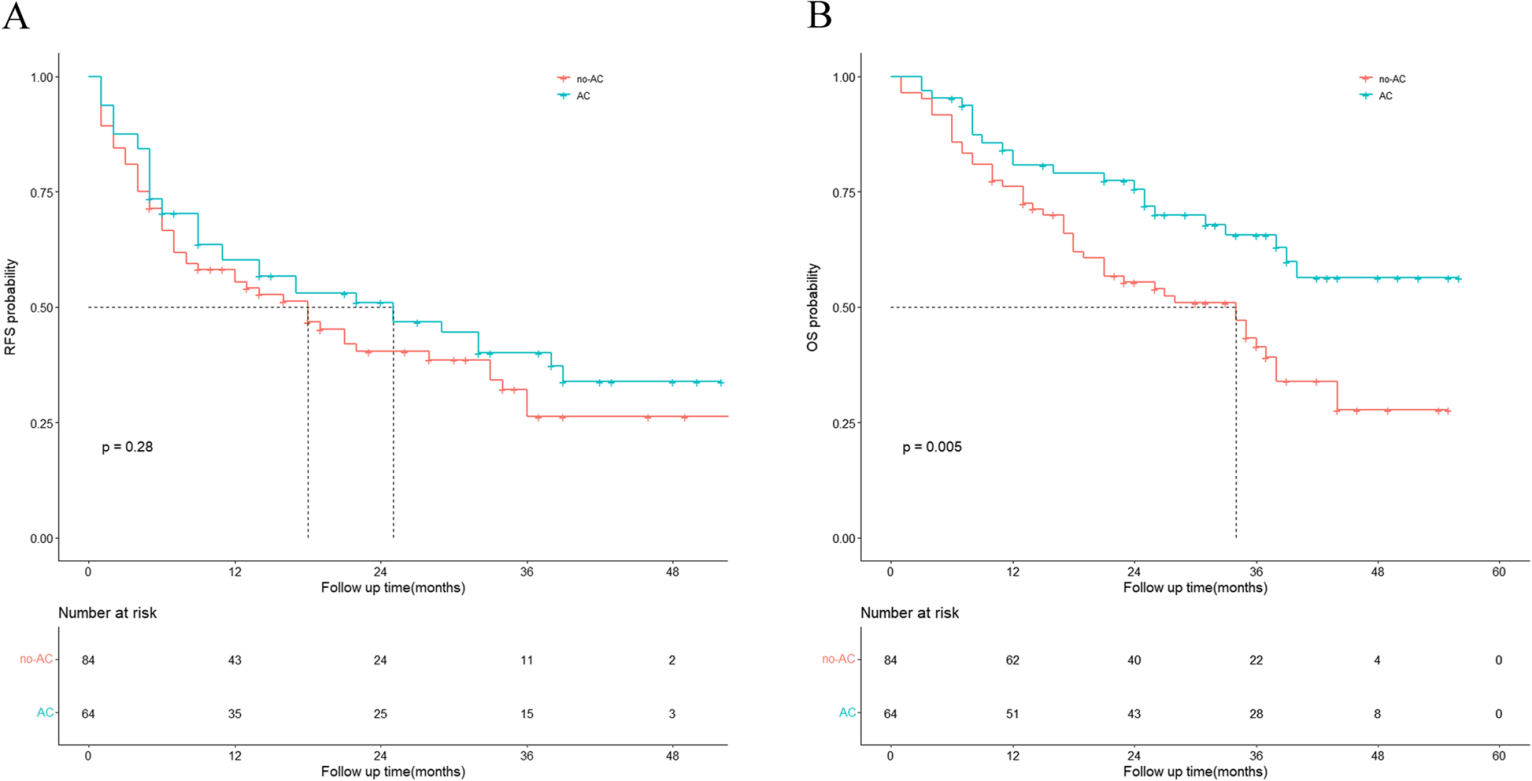
Kaplan-Meier analyses for RFS (**A**) and OS (**B**) based on postoperative adjuvant chemotherapy

The median RFS was 18 months in the non-AC group and 25 months in the AC group. The median OS was 34 months in the non-AC group; however, it was not reached in the AC group. RFS was not significantly different between the two groups (*P* = 0.28), whereas OS was significantly better in the AC group than in the non-AC group (*P* = 0.005). The Kaplan–Meier survival curve is shown in Fig. 2.

### Prognostic factors of overall survival

Based on univariate analysis, four clinical factors were identified as prognostic factors for OS (Table 2). Subsequently, Cox regression analysis was performed to screen for independent prognostic factors, which revealed that nerve invasion (hazard ratio [HR], 2.66; 95% confidence interval [CI], 1.50–4.73; *P* = 0.001), preoperative elevation of cancer antigen 19–9 (CA 19–9) levels (HR, 1.94; 95% CI 1.18–3.20; *P* = 0.009), and postoperative adjuvant chemotherapy (HR, 0.51; 95% CI 0.31–0.84; *P* = 0.009) resulted in significantly different OS (Table 2).

**Table 2:** Univariate and multivariate analysis of overall survival in a whole cohort

Characteristics	Univariate analyses			Multivariate analyses		
	HR	CI95	***P***.value	HR	CI95	***P***.value
Age	1.18	0.7–1.98	0.533			
Gender	1.07	0.67–1.69	0.790			
Tumor differentiation	0.73	0.45–1.18	0.201			
Tumor size	1.43	0.89–2.31	0.142			
Microvascular invasion	1.46	0.91–2.34	0.121			
Nerve invasion	2.71	1.55–4.76	**0.000**	2.66	1.5–4.73	**0.001**
Preoperative CA199 > 37.00u/ml	1.86	1.14–3.02	**0.013**	1.94	1.18–3.2	**0.009**
Preoperative TBIL> 17.1 umol/L	1.19	0.62–2.26	0.600			
HbsAg positive	0.95	0.6–1.52	0.843			
Surgical type	0.51	0.29–0.9	**0.021**	0.63	0.35–1.13	0.124
Postoperative targeted therapy	0.9	0.5–1.62	0.734			
Postoperative adjuvant chemotherapy	0.5	0.3–0.82	**0.006**	0.51	0.31–0.84	**0.009**

### Survival analysis of the AC (three chemotherapy regimens) and non-AC groups

**Fig. 3:**
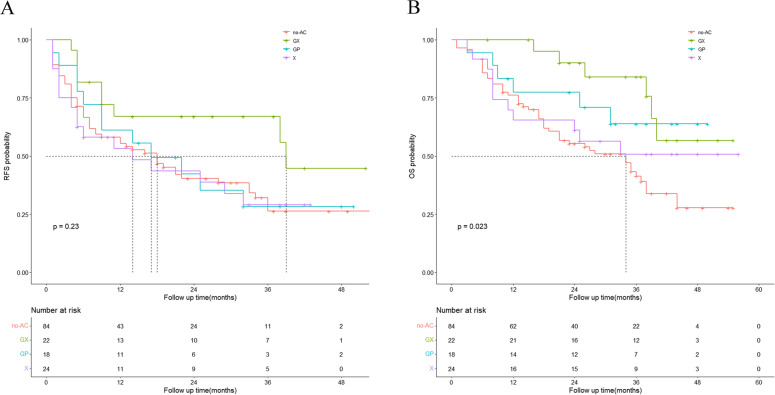
Kaplan-Meier analyses for RFS (**A**) and OS (**B**) based on different chemotherapy regimens

**Fig. 4:**
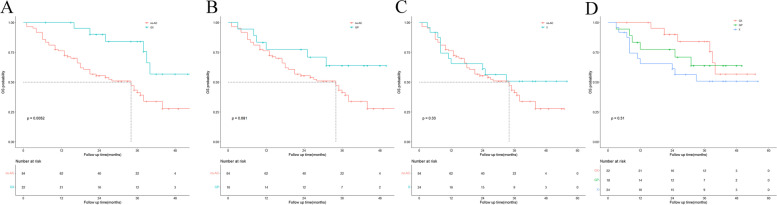
Kaplan-Meier analyses for OS based on different chemotherapy regimens. **A** OS in the no-AC group versus the GX group; **B** OS in the no-AC group versus the GP group; **C** OS in the no-AC group versus the X group; **D** The comparisons of OS among GX group, GP group and X group

The median RFS of the GX, GP, and X groups was 39, 17, and 14, respectively; however, the median OS was not reached in the three groups. The RFS rate was not different between the GX, GP, and X groups and the non-AC group (*P* = 0.23), whereas the OS rate was significantly different between the GX, GP, and X groups and the non-AC group (*P* = 0.023). The Kaplan–Meier curve is shown in Fig. 3. The OS rate of the GX group was significantly better than that of the non-AC group (*P* = 0.0052) but was not significantly different between the GP and X groups and the non-AC group (*P* = 0.081, *P* = 0.33). Moreover, no significant difference was observed in the OS rate among the GX, GP, and X groups (*P* = 0.31). The Kaplan-Meier curve is shown in Fig. 4.

### Survival analysis of patients with elevated preoperative CA 19–9 levels and those with pathological nerve invasion

**Fig. 5:**
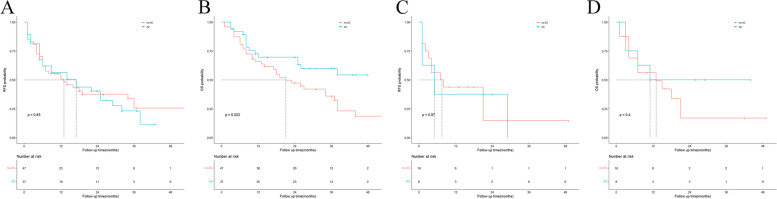
Kaplan-Meier analyses for RFS and OS based on postoperative adjuvant chemotherapy. A,RFS in the no-AC group versus the AC group in patients with preoperative CA19–9 levels more than 37 U/ml;B,OS in the no-AC group versus the AC group in patients with preoperative CA19–9 levels more than 37 U/ml;C,RFS in the no-AC group versus the AC group in patients with pathological nerve invasion;D,OS in the no-AC group versus the AC group in patients with pathological nerve invasion

Among patients with preoperative CA 19–9 levels of > 37 U/mL, RFS was not different between the non-AC and AC groups (*P* = 0.83); however, OS was better in the AC group that in the non-AC group (*P* = 0.022). Among patients with nerve invasion, RFS and OS were not different between the non-AC and AC groups (*P* = 0.97, *P* = 0.4). The Kaplan–Meier survival curve is shown in Fig. 5.

### Evaluation of the safety of postoperative adjuvant chemotherapy

A total of 64 patients with early-stage ICC received postoperative adjuvant chemotherapy for safety analysis, and all adverse events were evaluated. Of these 64 patients, 53 (82.8%) had varying degrees of adverse reactions. The incidence of aspartate aminotransferase (AST) elevation (*n* = 48; 75.0%) was highest, followed by nausea (*n* = 44; 68.8%). In the GX, GP, and X groups, patients were relieved of grade 1/2 adverse reactions after symptomatic treatment, whereas the condition of patients with grade 3–4 adverse reactions improved after drug dosage reduction, drug withdrawal, and symptomatic treatment. In the GX group, 1 patient was relieved of immune myositis after hormone shock therapy. In the GP group, 1 patient had a severe rash that improved after the administration of oral loratadine and intramuscular diphenhydramine for 1 week. The incidence of grade 3–4 adverse reactions was < 18.2% in the three groups, and no death owing to such reactions occurred in any group (Table 3).

**Table 3:** Postoperative adjuvant chemotherapy related adverse reactions

AE	ALL (***n*** = 64)		GX groups (***n*** = 22)		GP groups (***n*** = 18)		X groups (***n*** = 24)	
	Grade 1–2	Grade 3–4	Grade 1–2	Grade 3–4	Grade 1–2	Grade 3–4	Grade 1–2	Grade 3–4
Nausea	44(68.8%)	0	15(68.2%)	0	14(77.8%)	0	15(62.5%)	0
Diarrhea	31(48.4%)	3(4.7%)	10(45.5%)	0	8(44.4%)	0	13(54.2%)	3(12.5%)
Rash	28(43.8%)	0	10(45.5%)	0	7(38.9%)	0	11(45.8%)	0
Hand-foot syndrome	25(39.1%)	0	11(50.0%)	0	5(27.8%)	0	9(37.5%)	0
Fatigue	30(46.9%)	5(7.8%)	11(50.0%)	0	7(38.9%)	3(16.7%)	12(50.0%)	2(8.3%)
Leucopenia	16(25.0%)	7(10.9%)	9(40.9%)	4(18.2%)	5(27.8%)	1(5.6%)	2(8.3%)	2(8.3%)
Anemia	30(46.9%)	4(6.3%)	11(50.0%)	4(18.2%)	11(61.1%)	0	8(33.3%)	0
Thrombocytopenia	28(43.8%)	2(3.1%)	14(63.6%)	1(4.5%)	7(38.9%)	1(5.6%)	7(29.2%)	0
ALT elevation	35(54.7%)	0	15(68.2%)	0	8(44.4%)	0	12(50.0%)	0
AST elevation	48(75.0%)	0	16(72.7%)	0	11(77.8%)	0	18(75.0%)	0
TBIL elevation	32(50.0%)	0	16(72.7%)	0	7(38.9%)	0	9(37.5%)	0
Proteinuria	31(48.4%)	2(3.1%)	9(40.9%)	2(9.1%)	8(44.4%)	0	14(58.3%)	0
Hematuresis	21(32.8%)	2(3.1%)	10(45.5%)	2(9.1%)	6(33.3%)	0	5(20.8%)	0
Creatinine elevation	30(46.9%)	0	12(54.5%)	0	6(33.3%)	0	12(50.0%)	0

## Discussion

At present, surgery is the mainstay of treatment for patients with ICC; however, owing to the high malignancy and heterogeneity of ICC, the postoperative recurrence and mortality rates are high, and the postoperative 5-year OS rate is only 25–40% [4].

Although some studies [12] have reported that postoperative adjuvant chemotherapy does not benefit the survival of patients with ICC, the BILCAP study [13], CSCO expert consensus on the diagnosis and treatment of biliary system tumors (2019 edition) [14], and ASCO [5] recommend that patients with cholangiocarcinoma should receive capecitabine as adjuvant therapy postoperatively. However, the selection of patients eligible for adjuvant chemotherapy remains controversial. A multicenter retrospective analysis showed that postoperative adjuvant chemotherapy can prolong the survival of patients with a high risk of recurrence (patients with T2, T3, T4, and N1 disease) [8]. A consensus reached by surgical treatment experts in 2020 recommends that postoperative adjuvant radiotherapy and chemotherapy can be used for patients with ICC with R1 resection, N1-stage disease, or large vessel invasion [15]. Moreover, many researchers have proposed that postoperative adjuvant chemotherapy should be selected for patients with a high risk of recurrence, such as patients with >T2-stage disease, R1 resection, lymph node metastasis, vascular invasion, and nerve infiltration [11, 16]. In this study, the relevant clinical data of patients with T1N0M0-stage ICC who underwent radical (R0) resection were collected, and the patients were divided into the chemotherapy (AC) and non-chemotherapy (non-AC) groups. No significant difference was observed in baseline data between the two groups. In addition, the results of survival analysis showed that the RFS rate was not different between the two groups (*P* = 0.28), whereas the OS rate of the AC group was significantly better than that of the non-AC group (*P* = 0.005). These results suggest that postoperative adjuvant chemotherapy is significantly beneficial for patients with T1N0M0-stage ICC after radical resection. Similarly, Wang et al. [17] conducted a multicenter retrospective analysis involving 412 patients with ICC and suggested that patients aged ≤50 years and with normal CA 19–9 levels, good tumor differentiation, no subfoci, and no vascular invasion can benefit from adjuvant chemotherapy. We speculate that patients with T1N0M0-stage disease can benefit from postoperative adjuvant chemotherapy because of its killing effect on early microscopic metastases in the liver, blood, or lymph nodes. In addition, some studies have attributed the benefits of adjuvant chemotherapy to the better physical condition and chemotherapy tolerance of patients with early-stage tumors and the higher completion rate of adjuvant chemotherapy [18].

However, the choice of different postoperative adjuvant chemotherapy regimens for ICC remains controversial. To date, BILCAP [13] is the only phase III randomized controlled study with positive results. Compared with the observation group, all patients with biliary tract carcinoma (BTC) treated with capecitabine had improved OS. Although the beneficial effects were not significant according to the intention-to-treatment analysis, the capecitabine group achieved an absolute survival benefit of 14.7 months. Based on the results of the BILCAP trial, the ASCO (2019) clinical practice guidelines [5] recommend capecitabine for 6 months of adjuvant therapy. However, PRODIGE-12 [12], a phase III randomized controlled study, showed that gemcitabine combined with oxaliplatin adjuvant chemotherapy did not improve postoperative RFS and OS among patients with BTC. Furthermore, the phase III Bile Cancer Adjuvant Trial (BCAT) [19] showed that adjuvant gemcitabine was not associated with improved RFS or OS. The median RFS for the gemcitabine-treated and observation groups was 36.0 and 39.9 months (*P* = 0.69), respectively, whereas the median OS for the gemcitabine-treated and observation groups was 62.3 and 63.8 months (*P* = 0.96), respectively. In a phase II study, Siebenhuner et al. [20] reported that no significant differences were observed in the median DFS (14.4 months versus 28.8 months, respectively; *P* = 0.22) and OS (46.9 months versus 36.9 months, respectively; *P* = 0.67) between patients treated with gemcitabine monotherapy and those treated with the combination of gemcitabine and cisplatin. In this study, the results of subgroup analysis showed that the OS of patients who received the combination of gemcitabine and capecitabine (GX group) was significantly better than that of patients who underwent only surgical treatment (non-AC group) (undefined OS versus 34 months, respectively; *P* = 0.0052). However, the OS of patients treated with the combination of gemcitabine and cisplatin (GP group) and capecitabine monotherapy (X group) was not significantly different from that of the non-AC group (undefined OS versus 34 months, respectively, *P* = 0.081; undefined OS versus 34 months, respectively, *P* = 0.33). The median OS of all patients treated with the three adjuvant chemotherapy regimens was better than that of patients in the non-AC group; however, only the combination of gemcitabine and capecitabine achieved statistical significance, suggesting that combination therapy with gemcitabine and capecitabine may be more effective than capecitabine monotherapy and combination therapy with gemcitabine and cisplatin for early-stage ICC. This finding is different from that of the BILCAP study, in which capecitabine monotherapy showed good efficacy [13]. This discrepancy may be attributed to the very different subjects of the two studies. We believe that there are two main reasons for this difference:1) A large proportion of patients in the BILCAP study [13] had a high risk of recurrence (N1, 47.0%; R1 resection, 37.6%), whereas all subjects in this study had a low risk of recurrence (N0, 100%; R0 resection, 100%);2) The adjuvant chemotherapy regimen in this study is different from that in the BILCAP study [13]. There are three chemotherapy regimens in this study (gemcitabine + capecitabine, gemcitabine + cisplatin, and capecitabine), while only one chemotherapy regimen was used in the BILCAP study [13] (capecitabine). Different chemotherapy regimens may have a great impact on the OS of patients in the two studies. In this study, combination therapy with gemcitabine and capecitabine had the best therapeutic effect. In this regard, our interpretation is that there is no absolutely excellent adjuvant chemotherapy regimen for cholangiocarcinoma at present, and the most appropriate chemotherapy regimen may be different for patients in different regions and clinical states. For patients included in this study, gemcitabine plus capecitabine may be more suitable for them. In terms of safety, the incidence of grade 3–4 adverse reactions among patients treated with the three adjuvant chemotherapy regimens was < 18.2%, without the occurrence of death owing to adverse drug reactions. In a study by Siebenhuner et al. [20], neutropenia (57%), leukopenia (38%), thrombocytopenia (19%), and fatigue (19%) were the most common grade 3 or 4 adverse events among patients treated with gemcitabine plus cisplatin. In another phase II study (UMIN000001294), neutropenia (27%), anemia (17%), and leukopenia (14%) were the most common grade 3 or 4 adverse events in 72% of patients who had completed planned adjuvant therapy. In the BILCAP trial [13], hand–foot syndrome (20%), diarrhea (8%), and fatigue (8%) were the most common grade 3 or 4 adverse events among patients treated with capecitabine monotherapy. The findings of the abovementioned studies are similar to those of this study, indicating that adjuvant chemotherapy is relatively safe for patients with ICC.

CA 19–9 is an important clinical marker (normal levels < 37 U/mL) for pancreatic cancer, which is also of great significance in the identification and efficacy evaluation of bile duct tumors [21, 22]. IT is highly sensitive for the diagnosis of ICC; however, when benign diseases such as biliary calculi cause obstructive jaundice, CA 19–9 secreted by bile duct epithelial cells can flow back into the blood and lead to false-positive results [23]. In this study, multivariate Cox regression analysis showed that elevated CA 19–9 levels (*P* = 0.034) were an independent risk factor for the poor prognosis of patients with ICC with R0 resection. Similarly, many studies have reported that high preoperative levels of CA 19–9 are important for the prognosis of ICC in clinical settings [24, 25]. Nerve invasion is a common finding in postoperative pathological examination of ICC. Shirai et al. [26] found that 80% of patients with ICC had nerve invasion. In addition, a multicenter study [27] suggested that nerve invasion was associated with poorer OS among patients with ICC and should be used as a standard for adjuvant chemotherapy. In this study, multivariate Cox regression analysis showed that nerve invasion (*P* = 0.001) was an independent risk factor for the poor prognosis of patients with T1N0M0-stage ICC with R0 resection, which is consistent with the results of several studies [28, 29]. In this study, jaundice occurred in some patients (13 in the non-AC group and 5 in the AC group). Intrahepatic cholangiocarcinoma usually causes obstructive jaundice due to tumor compression of the porta hepatis. Nicolas Golse et al. [30] found that the incidence of liver dysfunction after hepatectomy is higher if the patient is jaundiced for a long time before surgery. However,there is still a great deal of controversy about the level of total bilirubin before liver resection. Our experience is that total bilirubin usually needs to be less than 100 umol/L. This is for the faster recovery of postoperative liver function, also for the safety of postoperative patients. However, this was a small-sample single-center retrospective study. In the future, large-sample and multicenter studies should be conducted to guide the selection of patients eligible for adjuvant chemotherapy and develop different adjuvant chemotherapy regimens.

## Conclusion

Adjuvant chemotherapy can prolong the OS of patients with T1N0M0-stage ICC who have received surgical treatment, and preoperative elevation of CA 19–9 levels and nerve invasion are independent risk factors for the poor prognosis of these patients. Combination therapy with gemcitabine and capecitabine, combination therapy with gemcitabine and cisplatin, and capecitabine monotherapy are safe for patients with T1N0M0-stage ICC.

## Data Availability

The data used to support the findings of this study are included within the article.

## Abbreviations

ICC: Intrahepatic cholangiocarcinoma
RFS: Relapse-free survival
OS: Overall survival
AC: Adjuvant chemotherapy
GX: Gemcitabine+capecitabine
GP: Gemcitabine+cisplatin
X: Capecitabine monotherapy
CA 19–9: Cancer antigen 19–9
